# Absence of Flexor Carpi Radialis during an Elective Carpometacarpal Arthroplasty of the Thumb: A Rare Anatomical Variation

**DOI:** 10.1155/2016/7853487

**Published:** 2016-03-14

**Authors:** Stratos S. Sofos, Muhammad Riaz

**Affiliations:** ^1^Whiston Hospital, Warrington Road, Prescot L35 1DR, UK; ^2^Castle Hill Hospital, Castle Road, Hull HU5 3EU, UK

## Abstract

*Purpose*. We present an extremely rare anatomical variation of unilateral flexor carpi radialis (FCR) absence. This rare anatomical variation posed a clinical dilemma to us and we highlight the importance of the surgeon being aware of this anatomical variation of an important structure both as a reconstruction tool and as an anatomical landmark.* Methods*. This anatomical variation of the unilaterally absent FCR was found upon dissection during a carpometacarpal arthroplasty of the thumb.* Results*. Upon the discovery of an absent FCR tendon, we proceeded with a simple trapeziectomy.* Conclusions*. We present an extremely rare anatomical variation of unilateral FCR absence. This rare anatomical variation may pose clinical dilemmas to the operating surgeon who aims to utilise the FCR either for tendon transfer, for tendon graft, or, as seen in our case, in the reconstruction of a carpometacarpal excision at the thumb. We highlight this diagnosis of suspicion, which may influence the clinical procedure.

## 1. Background

Absence of flexor carpi radialis (FCR) in a clinical setting is rare, having identified only one other case in the English literature [1]. We present a case where FCR was absent during an elective carpometacarpal (cmc) arthroplasty of the left thumb, and we discuss this anatomical variation.

## 2. Case Report

**Figure 1:**
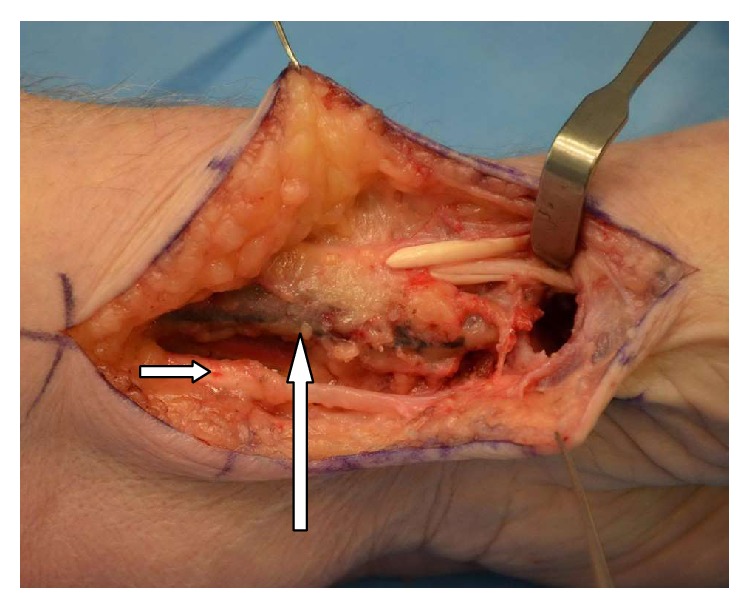
Radial artery marked by vertical arrow and FCR not visible as expected ulnar to the radial artery. Palmaris Longus seen inferiorly by horizontal arrow in the photo and APL and EPB superiorly.

We present the case of 75-year-old lady, who attended our outpatients' clinic with a painful left thumb secondary to osteoarthritis at the CMC joint. A trapeziectomy using an FCR ligament reconstruction under general anaesthesia was discussed and agreed upon preoperatively. However, intraoperatively it was noted that FCR was absent (Figure 1), thus changing our clinical approach.

**Figure 2:**
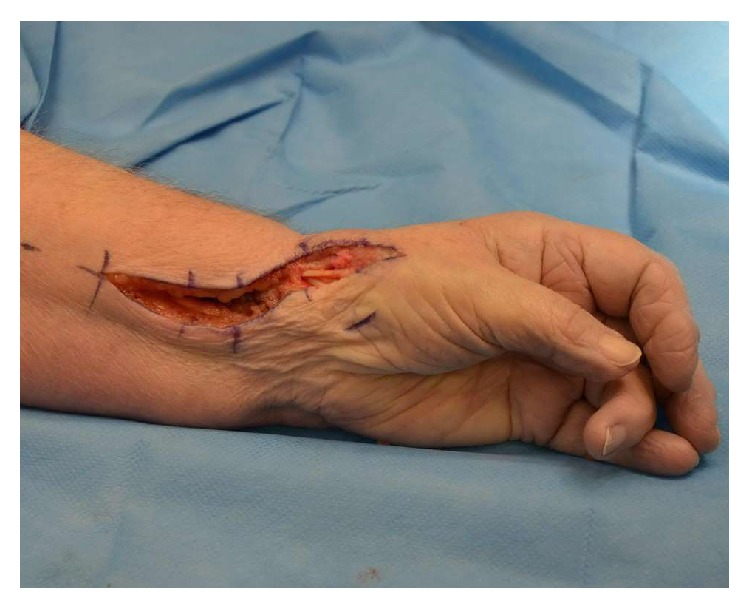
Surgical incision for CMC joint arthroplasty of left thumb.

**Figure 3:**
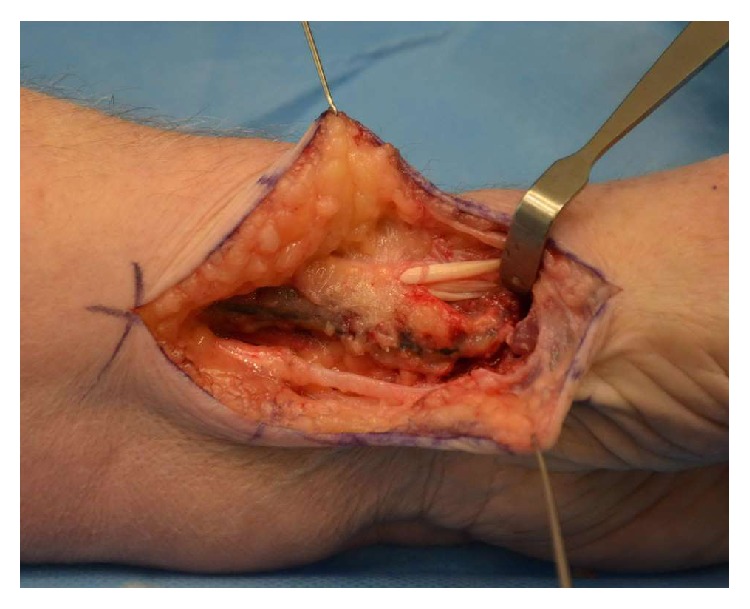
APL and EPB retracted. Radial artery seen inferior to the tendons.

A standard approach was taken to CMC arthroplasty starting with an incision on the radial border of the thenar eminence over the first metacarpal border approximately 2 cm distal to the CMC joint curving toward the flexor carpi radialis (FCR) tendon at the wrist (Figure 2). The subcutaneous tissue was dissected, taking care of preserving the branches of the superficial radial nerve. The abductor pollicis longus (APL) and the extensor pollicis brevis (EPB) are identified and protected (Figure 3).

The radial artery was identified in the proximal aspect of the wound overlying the trapezium. It was mobilized proximally and protected.

FCR normally lies radial to the Palmaris Longus (PL) at the level of the wrist and enters a fibroosseous tunnel just proximal to the trapezium. It is inserted anteriorly to the base of the 2nd metacarpal, partially to the trapezial tuberosity and partially to the base of the third metacarpal. However, it was absent from our patient's left wrist. The patient had a subsequent ultrasound scan (USS) on the contralateral, right wrist, which confirmed presence of FCR. As per the clinical history, the patient did not have any history of trauma. In addition, there were no functional problems reported by the patient of the affected hand compared to the contralateral side, which did contain an FCR.

To complete the operation manual traction was applied to the thumb to identify the CMC joint. The capsule was split from the trapezial metacarpal joint to the scaphotrapezial joint longitudinally. The capsule was then subperiosteally elevated off the trapezium and the trapezium was removed.

Given this unexpected anatomical variation, we decided on converting the operation to a “simple trapeziectomy” as opposed to using different reconstructive methods, since the patient had not formally consented to them. We reviewed the patient in our outpatients' clinic postoperatively and she had made an unremarkable recovery.

## 3. Discussion

Muscles in the upper limb originate from somites, and every muscle is identifiable by week 7 of the embryological development. The muscles differentiate from superficial to deep one, with the flexor digitorum profundus, flexor carpi radialis, and flexor carpi ulnaris splitting off first, followed by the flexor pollicis longus. Tendons develop independently and are derived from limb bud mesoderm rather than from somites. In fact, tendons develop in the absence of muscle, but they disintegrate later [1].

Anatomical variations of FCR have been noted in anatomical studies by Bergman et al. [2] with regard to its origin and insertion or having an additional slip from other muscles. There was only one case we identified with clinical relevance to the absence of FCR [3], related to tendon transfer in a child.

In our case, the absence of FCR had clinical and ethical implications, which influenced our management, since alternate options for reconstructions were possible.

Three of the most common treatment methods for a thumb CMC arthroplasty are as follows. (a) The first one is simple trapeziectomy, where the defect is left unrepaired. The advantages of a simple trapeziectomy are that it is simple and fast [4]. There is a potential for instability and loss of trapezial height; however, we know from the literature [5] that there are no statistically significant differences in postoperative grip strength, tip pinch strength, and key pinch strength between a tendon reconstruction and simple trapeziectomy. (b) The second option is complete trapeziectomy with FCR ligament reconstruction with no interposition.

In this technique, the entire trapezium is excised with preservation of a dorsally and distally based capsular flap. Variations to this technique exist, with some utilising Kirschner wires for additional stability.

(c) Trapezial resection suspensionplasty with abductor pollicis longus tendon is the third commonly used procedure. With this technique the entire dorsal slip of the APL tendon is used for the ligament reconstruction. There are variations to this technique reported also.

## 4. Summary

We present an extremely rare anatomical variation of unilateral FCR absence. This rare anatomical variation may pose clinical dilemmas to the operating surgeon who aims to utilise the FCR either for tendon transfer, for tendon graft, or, as seen in our case, in the reconstruction of a carpometacarpal excision at the thumb. We highlight this diagnosis of suspicion, which may influence the clinical procedure.
